# Nutritional Practices During the Transition to Motherhood: A Systematic Qualitative Review

**DOI:** 10.3390/nursrep16070234

**Published:** 2026-07-06

**Authors:** Artemisia Kokkinari, Maria Dagla, Kleanthi Gourounti, Evangelia Antoniou, Georgios Iatrakis

**Affiliations:** 1 Department of Midwifery, School of Health & Care Sciences, University of West Attica, 12243 Egaleo, Greece; mariadagla@uniwa.gr (M.D.); kgourounti@uniwa.gr (K.G.); lilanton@uniwa.gr (E.A.); giatrakis@uniwa.gr (G.I.); 2 Laboratory of Midwifery Care during Antenatal and Post Natal Period-Breastfeeding—L.M.C.A.P.-BF, 12243 Egaleo, Greece

**Keywords:** maternal nutrition, transition to motherhood, qualitative research, food practices, maternal identity, self-care, autonomy, mental health

## Abstract

**Background:** The transition to motherhood represents a critical life phase marked by profound biological, psychological and social changes. During this period, women’s nutritional practices are shaped not only by physiological needs but also by shifting identities, caregiving responsibilities and social expectations. Although nutrition during pregnancy and the postpartum period has been widely studied from a biomedical perspective, less attention has been paid to how women experience, negotiate and attribute meaning to food during the transition to motherhood. **Objective:** This systematic qualitative review aimed to synthesise existing qualitative evidence on women’s experiences of nutritional practices during the transition to motherhood, with particular attention to food as self-care, control, autonomy, identity formation and mental well-being. **Methods:** A systematic search of electronic databases was conducted to identify qualitative studies exploring women’s experiences of nutrition during pregnancy and early motherhood. Eligible studies employed qualitative methodologies such as interviews, focus groups or ethnographic approaches. Study selection followed PRISMA guidelines. Methodological quality was appraised using established qualitative appraisal tools. A thematic synthesis approach was used to integrate findings across studies. **Results:** The synthesis identified several interrelated themes: nutrition as a form of self-care and emotional regulation; loss of autonomy and heightened moral surveillance around food choices; food practices as a means of performing and negotiating “good motherhood”; and the emotional burden of dietary expectations in relation to mental health and identity. Women described navigating competing demands between their own nutritional needs and those of their infants, often within contexts of social judgement and limited support. **Conclusions:** Nutritional practices during the transition to motherhood extend beyond health behaviours and are deeply embedded in issues of identity, autonomy and care. Recognising the social and emotional dimensions of maternal nutrition may inform more holistic, woman-centred approaches to nutritional guidance and maternal health support.

## 1. Introduction

The transition to motherhood represents a profound biographical shift, encompassing significant changes in women’s bodies, identities, social roles and everyday practices. During this period, nutrition has traditionally been approached through biomedical and public health lenses, with an emphasis on nutrient requirements, dietary adequacy and risk reduction for maternal and infant outcomes. While such approaches are fundamental to maternal health, they tend to prioritise measurable dietary behaviours, often overlooking how women experience, interpret and negotiate food in their daily lives during pregnancy and early motherhood.

Food is not solely a source of biological nourishment but also a socially and culturally embedded practice through which care, responsibility and moral values are negotiated. It is also a social, cultural and symbolic practice through which care, responsibility and moral values are expressed and contested. For women transitioning to motherhood, eating practices may operate simultaneously as forms of self-care, strategies for maintaining control, and sites of tension where personal needs intersect with social expectations of maternal responsibility. Within these contexts, nutrition can become subject to heightened surveillance and moral evaluation, shaping women’s experiences of autonomy, guilt and self-regulation. Recent qualitative research further suggests that women may experience increasing sociocultural pressure surrounding body image, weight regulation and dietary behaviours during pregnancy and the postpartum period, particularly in relation to expectations of responsible motherhood [1].

From a psychosocial perspective, the transition to motherhood is also a critical period of identity reconstruction. As women renegotiate their sense of self in relation to caregiving roles, food and eating practices may play an important role in performing and sustaining notions of “good motherhood,” while also reflecting losses of personal agency, time and self-focused care. These processes are increasingly recognised as closely linked to maternal emotional well-being and mental health.

Although a growing body of qualitative research has explored aspects of food, eating and health during pregnancy and early motherhood, this literature remains fragmented across disciplines and perspectives. A comprehensive synthesis of women’s lived experiences of nutritional practices during the transition to motherhood is therefore lacking. Addressing this gap is essential for developing more holistic and woman-centred approaches to maternal nutrition that recognise food not only as a health behaviour, but also as a social, emotional and identity-related practice.

### 1.1. Nutrition During the Transition to Motherhood: From Biomedical Focus to Lived Experience

Nutrition during pregnancy and early motherhood has been extensively examined within biomedical and public health frameworks, primarily focusing on nutrient intake, dietary adequacy, gestational weight gain and maternal–infant outcomes [2,3,4]. Large-scale epidemiological studies and clinical guidelines have emphasised optimal dietary patterns as a means of reducing adverse pregnancy outcomes and supporting infant development [3,5]. While this body of research has contributed significantly to maternal and child health, it has been criticised for adopting a predominantly reductionist view of nutrition, framing food primarily as a vehicle for nutrients rather than as a socially embedded practice [6].

Qualitative scholars have argued that such biomedical approaches often fail to capture how women actually experience and negotiate food in their everyday lives during the transition to motherhood [7,8]. Lupton [7], for example, demonstrated that women’s engagement with food during pregnancy is shaped not only by health advice but also by emotions, moral meanings and social expectations. Similarly, DeVault’s seminal work on feeding and gender highlighted that food practices are deeply intertwined with caring labour and gendered responsibilities, long before women become mothers [9]. These insights suggest that nutrition during the transition to motherhood cannot be fully understood without attending to women’s subjective experiences and the social contexts in which food practices occur.

### 1.2. Food as Self-Care During Pregnancy and Early Motherhood

A growing body of research has highlighted that food and eating practices may function as forms of self-care during pregnancy and early motherhood [10,11,12]. For example, Hauff et al. [10] found that women often described eating well as a way of supporting their physical and emotional wellbeing during pregnancy, particularly in the context of bodily changes and pregnancy-related discomfort. Food was frequently framed as a source of comfort, stability and reassurance during a period characterised by uncertainty.

However, the meaning of self-care through nutrition appears to change following childbirth. While pregnancy often encourages women to pay closer attention to their dietary intake, the postpartum period is frequently characterised by disrupted eating patterns and reduced prioritisation of maternal nutrition, as infant care and household responsibilities take precedence [11,12,13].

Public health messages and social norms surrounding “eating for two” and appropriate weight gain during pregnancy can reinforce the idea that maternal food choices are subject to scrutiny and evaluation [14]. Research also indicates that pregnant women actively seek nutrition-related information and often feel a strong sense of responsibility for making the “right” dietary decisions, which can further intensify perceived pressure around food choices [15]. Women have also described a need for clearer, more individualised nutritional support during pregnancy, particularly when attempting to navigate multiple and sometimes inconsistent sources of dietary information [15,16]. Qualitative evidence suggests that these expectations may shape how women interpret their own behaviours and evaluate their competence as mothers.

### 1.3. Control, Surveillance and Loss of Autonomy in Maternal Food Practices

The transition to motherhood has also been identified as a period in which women experience heightened control and surveillance over their bodies and behaviours, particularly in relation to food [7,17]. Lupton [7] described pregnancy as a “moral enterprise,” where women’s food choices are closely scrutinised by health professionals, family members and wider society. This surveillance often extends into the postpartum period, with mothers continuing to feel judged for their dietary practices, especially in relation to breastfeeding and infant feeding [18].

Qualitative evidence suggests that such scrutiny can lead to a perceived loss of autonomy over food choices. Research examining behavioural expectations surrounding weight management during pregnancy has also highlighted how health-related advice may be experienced as pressuring or difficult to negotiate, particularly among women who already feel subject to increased scrutiny regarding body weight and lifestyle behaviours [17]. Rather than empowering women, health messages were sometimes experienced as restrictive and anxiety-inducing. Studies of the postnatal period indicate that the everyday realities of caring for an infant, including fatigue, time scarcity and competing household demands, can further limit mothers’ opportunities to prioritise their own nutrition [12,19].

These experiences point to a tension between public health discourses that promote individual responsibility for “healthy eating” and women’s lived realities during the transition to motherhood, where control over food is often negotiated within structural, relational and emotional constraints.

### 1.4. Nutrition, Maternal Identity and the Performance of “Good Motherhood”

Food practices during the transition to motherhood are closely linked to the construction and performance of maternal identity. Qualitative and sociological research has shown that women often interpret feeding and nutrition-related practices as important indicators of being a “good mother” [7,19,20]. Lupton [7], for example, demonstrated that pregnant women frequently internalise public health discourses around food and risk, framing dietary regulation as part of their responsibility to protect the unborn child. Similarly, research on infant feeding has highlighted how women negotiate expectations of good motherhood through everyday decisions about care and nourishment [19].

Postnatally, these expectations may become embedded within broader cultural norms that emphasise intensive maternal responsibility. Studies have shown that contemporary motherhood ideals often position mothers as primarily responsible for managing children’s health and wellbeing, including feeding practices [20,21]. Within this framework, women’s own needs may be deprioritised, and deviations from recommended practices can be experienced as moral judgement or maternal inadequacy. Empirical research has also reported that mothers may feel guilt when relying on convenient food practices or when they perceive their behaviours as inconsistent with dominant expectations of maternal care [12]. In addition, research on pregnancy-related eating norms has highlighted how prevailing social expectations around maternal responsibility for nutrition can reinforce pressure on women to regulate their diets and conform to ideals of “appropriate” maternal behaviour [14].

These findings suggest that nutrition functions not only as a health behaviour but also as a symbolic practice through which women negotiate maternal identity, social legitimacy and moral worth.

### 1.5. Nutritional Practices and Maternal Mental Health

Emerging qualitative evidence highlights important links between nutritional practices, emotional well-being and mental health during the transition to motherhood [13,22,23]. Women have described how pressures to eat “correctly,” combined with fatigue and lack of support, contribute to stress, anxiety and feelings of inadequacy [13]. In a qualitative study, Faria-Schützer et al. [22] found that eating behaviours during the postpartum period were closely intertwined with women’s emotional wellbeing, as emotional distress and the challenges of adapting to motherhood influenced their dietary practices.

Furthermore, qualitative evidence suggests that the transition to motherhood is often accompanied by heightened self-expectations, perceived social judgement and emotional demands, all of which may contribute to maternal psychological burden [23]. Rather than being a neutral or supportive aspect of daily life, nutrition may become an additional site of pressure during a period already associated with increased vulnerability to mental health difficulties.

### 1.6. Rationale and Aim of the Review

Although qualitative research has increasingly examined aspects of food, diet and health during pregnancy and early motherhood, these studies are often located within different disciplinary traditions and research contexts. To date, there has been no systematic qualitative synthesis that integrates these findings to provide a comprehensive understanding of how women experience and attribute meaning to nutritional practices during the transition to motherhood. Recent qualitative research has increasingly highlighted the complex social, emotional and cultural dimensions of maternal food practices, yet the available evidence remains fragmented across populations, contexts and disciplinary perspectives [24].

While individual qualitative studies have provided valuable insights into maternal food practices, their findings remain dispersed across different cultural settings, populations and theoretical perspectives. As a result, it remains difficult to identify common patterns and broader conceptual understandings regarding the role of nutrition during the transition to motherhood. A qualitative synthesis is therefore needed to integrate this evidence and provide a more comprehensive interpretation of women’s experiences.

The aim of this systematic qualitative review is therefore to synthesise existing qualitative evidence on women’s experiences of nutritional practices during pregnancy and early motherhood, with a particular focus on self-care, control and autonomy, maternal identity, social expectations and mental well-being.

## 2. Materials and Methods

### 2.1. Study Design

This systematic qualitative review was conducted and reported in accordance with the Preferred Reporting Items for Systematic Reviews and Meta-Analyses (PRISMA 2020) statement.

The review process followed the PRISMA 2020 guidelines to ensure transparency and methodological rigor in study identification, screening, eligibility assessment, and inclusion.

A completed PRISMA checklist and flow diagram are provided as Supplementary Materials.

A thematic synthesis approach was employed to integrate findings from qualitative studies exploring women’s experiences of nutrition during the transition to motherhood.

The review protocol was not registered in a public database. No formal review protocol was prepared prior to the conduct of this systematic qualitative review.

### 2.2. Search Strategy

A comprehensive search strategy was developed to identify relevant qualitative studies. Electronic databases including PubMed, Scopus, Web of Science and CINAHL were searched using combinations of keywords related to motherhood, pregnancy, postpartum period, nutrition, food practices and qualitative research. Reference lists of included studies were also screened to identify additional relevant publications. Grey literature sources (e.g., theses, dissertations, institutional repositories and reports) were not searched, as the review aimed to synthesise evidence from peer-reviewed qualitative studies published in scientific journals. Study registries and ongoing research databases were not searched because the review focused exclusively on published qualitative studies.

Studies published between January 2010 and June 2026 were considered eligible in order to capture contemporary qualitative research on maternal nutrition and women’s experiences during the transition to motherhood. This timeframe was selected to reflect contemporary social, cultural and healthcare contexts influencing maternal nutrition, while also ensuring sufficient coverage of qualitative research conducted during a period characterised by substantial changes in maternity care, public health recommendations and digital sources of nutritional information. Earlier studies were therefore excluded, as they were considered less likely to reflect current experiences and healthcare environments relevant to contemporary motherhood and contemporary nursing and midwifery practice.

A full electronic search strategy was developed for each database in consultation with relevant guidelines for systematic reviews. The complete search string for PubMed is provided as an example to ensure reproducibility: (“Pregnancy”[MeSH] OR “Postpartum Period”[MeSH] OR pregnancy OR postpartum OR “transition to motherhood” OR “new mothers”) AND (“Nutrition”[MeSH] OR “Diet”[MeSH] OR nutrition OR diet OR “food practices” OR eating) AND (“Qualitative Research”[MeSH] OR qualitative OR interviews OR “focus groups” OR ethnograph*). Search strategies were adapted for use in other databases using appropriate controlled vocabulary and syntax. No restrictions on study design beyond qualitative methodology were applied. The full search strategies for all databases are provided in the Supplementary Materials.

### 2.3. Eligibility Criteria

Studies were included if they: employed qualitative or mixed-methods designs with qualitative components; explored women’s experiences of nutrition, food or eating practices; focused on pregnancy, the postpartum period or early motherhood; and were published in peer-reviewed journals in English.

For the purposes of this review, “early motherhood” was operationally defined as the period extending from childbirth to the first two years postpartum. This timeframe was selected because it reflects an important transitional phase during which women adapt to maternal caregiving roles, infant feeding practices, bodily changes, changes in daily routines, and evolving self-care responsibilities. This period is also widely recognised in maternal health research as a critical stage of adjustment and identity reconstruction following childbirth.

Studies were excluded if they were purely quantitative, focused exclusively on clinical dietary interventions without experiential data, or addressed eating disorders as the primary outcome. Eating disorders were not included because the review focused on women’s everyday nutritional experiences, food-related practices and meanings during pregnancy and early motherhood rather than on clinically diagnosed eating disorders, which constitute a distinct area of research with specialised psychological and psychiatric frameworks. Studies focusing exclusively on infant feeding practices, breastfeeding outcomes or child nutrition without exploring women’s own nutritional experiences were also excluded. Studies addressing breastfeeding, infant feeding, food insecurity, gestational weight gain, postpartum body image or family food practices were included only when they provided direct qualitative data on women’s own nutritional experiences, perceptions, behaviours or food-related practices during pregnancy or early motherhood.

### 2.4. Study Selection

Titles and abstracts were screened for eligibility, followed by full-text review of potentially relevant articles. Study selection was conducted systematically, with reasons for exclusion documented at each stage.

The screening process was conducted in two stages. All records were screened independently by two reviewers at both the title/abstract and full-text stages. Discrepancies were resolved through discussion, and where consensus could not be reached, a third reviewer was consulted. This process was implemented to minimise selection bias and enhance methodological rigor. The screening process was managed using a structured review approach consistent with PRISMA recommendations.

Study selection decisions were documented, and discrepancies were resolved through discussion.

**Figure 1:**
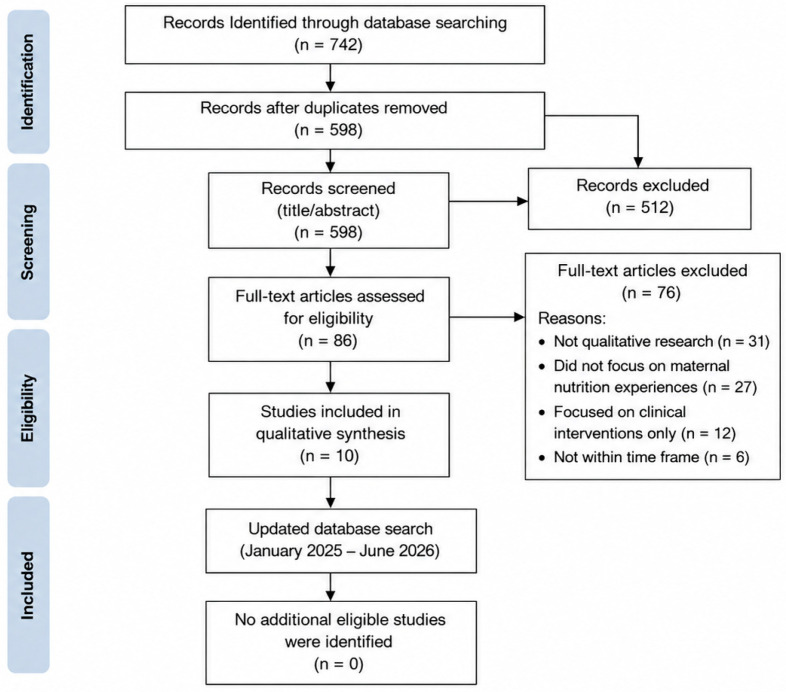
PRISMA flow diagram of study selection.

A total of 742 records were identified through database searching. After removing 144 duplicates, 598 records remained for title and abstract screening. Of these, 512 were excluded as they did not meet the inclusion criteria. Eighty-six full-text articles were assessed for eligibility, and 76 were excluded for reasons including lack of qualitative data, focus on clinical interventions, or not addressing maternal nutrition experiences. The excluded full-text articles covered a range of maternal and perinatal health topics. Most were excluded because they did not provide qualitative findings specifically addressing women’s experiences of nutrition during pregnancy or early motherhood, focused primarily on clinical dietary interventions, or employed quantitative study designs that did not meet the predefined eligibility criteria for inclusion in this qualitative synthesis. Ten studies met the inclusion criteria and were included in the final synthesis (Figure 1). An updated search covering the period from January 2025 to June 2026 was conducted prior to final manuscript submission in order to ensure the timeliness of the review. No additional studies meeting the eligibility criteria were identified during this update search. Therefore, the number of included studies and the overall synthesis remained unchanged.

### 2.5. Quality Appraisal

The methodological quality of included studies was assessed using the Critical Appraisal Skills Programme (CASP) checklist for qualitative research. Quality appraisal was used to inform interpretation of findings rather than to exclude studies. The quality appraisal was conducted by one reviewer using the CASP checklist and was subsequently reviewed by a second researcher to ensure consistency and accuracy. Discrepancies were discussed and resolved through consensus. The assessment was applied across all included studies to support the interpretation of findings. Detailed study-by-study CASP appraisal results are presented in Supplementary File S3.

### 2.6. Data Synthesis

Data extraction was conducted using a predefined and piloted data extraction form. The following variables were systematically extracted from each study: author(s), year of publication, country, study design, participant characteristics, data collection methods, and key qualitative findings related to nutritional practices. Data extraction was performed by one reviewer and independently checked by a second reviewer to ensure accuracy and consistency.

Data were synthesised using thematic synthesis. Data synthesis was informed by established approaches to qualitative evidence synthesis. Specifically, the analysis drew on principles of thematic synthesis, a method commonly used in systematic reviews of qualitative research to integrate findings across studies while preserving participants’ perspectives. This approach involves iterative coding of study findings, the development of descriptive themes, and the generation of higher-order analytical interpretations that extend beyond the results of individual studies. Thematic synthesis followed the approach proposed by Thomas and Harden, involving line-by-line coding, development of descriptive themes, and generation of analytical themes. This approach was selected as it is particularly suited to the synthesis of qualitative research, allowing for the integration of findings across studies while preserving the depth and context of participants’ experiences. This method has been widely applied in health research to synthesise qualitative evidence in a systematic and transparent manner [25]. The analysis was also informed by principles of thematic analysis as described in qualitative research methodology literature [26].

A standardized data extraction form was used to organise study characteristics and key qualitative findings from each included study. All relevant qualitative results were considered, including participant quotations and authors’ interpretations related to women’s experiences of nutritional practices, self-care, autonomy, identity, social expectations and emotional well-being. Where information was unclear or incomplete, it was interpreted based on the available description in the original studies. Prior to analysis, qualitative findings were organised into a consistent format to facilitate comparison across studies. This involved collating participant quotations and authors’ interpretations and ensuring that data were sufficiently detailed to support thematic coding. Following this, findings and participant quotations were extracted from each study and coded line by line. Codes were grouped into descriptive themes, which were then further interpreted to generate higher-order analytical themes capturing shared meanings across studies.

Reflexive discussions were conducted throughout the analytical process in order to enhance transparency and minimise potential interpretative bias. Throughout the coding process, emerging codes and preliminary themes were regularly reviewed and discussed among the research team. This process allowed alternative interpretations to be considered, challenged individual assumptions and helped minimise the influence of any single researcher’s perspective on theme development. Analytical decisions and theme development were discussed iteratively among the research team in order to enhance interpretative consistency and maintain transparency throughout the synthesis process.

As this review synthesised qualitative evidence, no quantitative effect measures were applied. Findings were analysed and presented as themes derived from participants’ experiences and authors’ interpretations.

All studies that met the predefined inclusion criteria were included in a single, overarching thematic synthesis. Eligibility for inclusion in the synthesis was determined based on alignment with the review’s focus on women’s experiences of nutritional practices during pregnancy and early motherhood.

The results of individual studies were summarised in tabular form (Table 1) to present key study characteristics. The findings of the synthesis were presented narratively through thematic categories, supported by examples from the included studies.

No formal sensitivity analyses were conducted, as the synthesis was based on qualitative data and aimed to generate interpretative themes rather than quantitatively pooled estimates. However, the consistency of themes across studies was considered during the analytical process to support the robustness of the findings. Importantly, these findings are also consistent with more recent qualitative research examining maternal nutrition, emotional wellbeing and caregiving practices across diverse socio-cultural settings [24,33,34,35,36]. These studies are cited as contextual literature to support the interpretation of the findings and were not included in the formal thematic synthesis. Due to the qualitative nature of the included studies and the use of thematic synthesis, formal assessment of statistical heterogeneity or subgroup analyses were not undertaken.

### 2.7. Assessment of Confidence in the Evidence

In line with established approaches to qualitative evidence synthesis, the strength of the evidence was considered throughout the analytical process. Particular attention was given to the consistency of findings across studies, the richness and depth of the data, and the methodological quality of included studies as assessed using the CASP checklist.

Although a formal GRADE-CERQual assessment was not conducted, the review aimed to enhance the credibility of the findings by systematically examining convergence and divergence across studies, as well as the extent to which themes were supported by data from multiple sources.

## 3. Results

These studies explored women’s experiences of nutrition during pregnancy and the early postpartum period across diverse socio-cultural contexts. The included studies represented a range of high-income and middle-income settings, including the United Kingdom, Canada, Australia, India, China and Taiwan. Sample sizes and participant characteristics varied across studies, but all focused on women’s lived experiences of food, diet or feeding practices during pregnancy or early motherhood.

Table 1 summarises the key characteristics of the ten qualitative studies included in this synthesis. The table provides details on study authors and year of publication, country, study design, participant characteristics, data collection methods, and the primary focus of each study. To further support interpretation of the diversity and transferability of the included evidence, Table 2 provides additional information on sample size, maternal stage, participant characteristics and cultural or contextual setting, where reported in the original studies. These studies collectively cover a range of socio-cultural contexts and capture women’s lived experiences of nutrition, food practices, and the transition to motherhood. While sample sizes and methodological approaches varied, all studies contribute insights into the themes of self-care, autonomy, identity, and the social and emotional dimensions of maternal nutrition.

Four overarching analytical themes were identified. Overall, the confidence in the identified themes ranged from moderate to high, reflecting consistency of findings across multiple studies despite some methodological limitations. Quality appraisal indicated that five studies were rated as high quality, three as moderate–high quality and two as moderate quality according to the CASP criteria. No studies were excluded on the basis of methodological quality, and the appraisal findings were used to inform the interpretation of the synthesis. The analytical themes were supported by data from multiple studies, with variations across contexts contributing to a richer understanding of women’s experiences of nutritional practices during the transition to motherhood. Several studies contributed to more than one analytical theme, reflecting the interconnected nature of women’s experiences of nutrition, care, identity and wellbeing during the transition to motherhood.

Some cross-cultural differences were observed across the included studies. Studies conducted in the United Kingdom, Canada and Australia frequently emphasised individual responsibility, autonomy and body image in relation to maternal nutrition. In contrast, studies from India, China and Taiwan highlighted the influence of family traditions, cultural dietary practices and collective expectations surrounding maternal and infant care. Despite these contextual differences, common themes relating to self-care, maternal responsibility, social expectations and emotional wellbeing were evident across settings.

### 3.1. Nutrition as Self-Care and Emotional Regulation

Across studies, nutrition during pregnancy was frequently framed by women as an important form of self-care and emotional regulation. Women frequently described paying closer attention to food choices during pregnancy as part of adapting to bodily changes and new emotional demands. For example, Blau et al. [27] reported that women discussed food cravings, dietary adjustments, and eating routines as strategies that helped them cope with pregnancy-related stress while also reinforcing a sense of care for the developing baby. In this context, eating practices were not only nutritional decisions but also part of how women navigated the physical and emotional experience of pregnancy. Hauff et al. [10] similarly found that food practices provided emotional reassurance and a sense of stability amid uncertainty, particularly for first-time mothers.

However, this framing shifted markedly in the postpartum period. Qualitative studies focusing on early motherhood suggest that once the baby is born, women’s own nutritional needs are often deprioritised within the demands of infant care. For instance, Bathula et al. [30] reported that postnatal women frequently described their eating patterns as shaped by caregiving routines, household dynamics, and culturally prescribed food practices. In many cases, meals became irregular or secondary to infant feeding and recovery, reflecting a broader transition from intentional dietary self-care during pregnancy to more pragmatic eating patterns in the early postpartum period. Participants often framed these changes as a normal and expected aspect of motherhood, emphasising the prioritisation of the baby’s needs over their own.

### 3.2. Loss of Autonomy, Surveillance, and Moral Regulation

A consistent finding across the included studies was women’s experience of diminished autonomy over their dietary choices. Olander et al. [8] highlighted that women encountered conflicting dietary advice from health professionals, family members, and online sources, leading to confusion and anxiety. Many women described feeling closely monitored and judged in relation to their food choices during pregnancy. Similar experiences of heightened self-regulation were reported in the qualitative study by Sui et al. [32], where pregnant women described monitoring their food intake closely in relation to expectations surrounding appropriate gestational weight gain. Participants frequently framed dietary control as a moral responsibility linked to protecting the developing baby, but also reported feelings of pressure and self-scrutiny when their eating practices did not align with perceived recommendations.

Marshall et al. [28] further illustrated how structural constraints, such as food insecurity, intensified this loss of control. Women described constant negotiation between nutritional ideals and material realities, often prioritising their children’s dietary needs over their own. These findings suggest that autonomy in maternal nutrition is not solely an individual matter but is deeply shaped by social, economic, and institutional forces.

### 3.3. Nutrition, Identity, and the Performance of “Good Motherhood”

Several studies emphasised the symbolic role of nutrition in the construction and performance of maternal identity. Murray-Davis et al. [29] found that women viewed postnatal dietary practices as evidence of their competence as mothers, particularly in relation to weight management and recovery of the pre-pregnancy body. Adherence to “healthy eating” norms was closely tied to perceptions of success and self-worth.

Cultural dimensions of this process were evident in the work of Bathula et al. [30], who demonstrated how traditional postpartum dietary beliefs shaped women’s experiences in India. Participants often complied with prescribed food rules imposed by family elders, even when these conflicted with personal preferences or biomedical advice. Nutrition thus functioned as a relational practice through which maternal identity was negotiated within family hierarchies.

### 3.4. Nutritional Practices and Maternal Mental Well-Being

Although mental health was not always the primary focus, several studies identified clear links between nutritional practices and emotional wellbeing. Faria-Schützer et al. [22] found that postpartum women often described eating behaviours as a response to emotional distress and the challenges of adapting to motherhood. Participants also reported neglecting their own nutritional needs while prioritising infant care and family responsibilities.

McLeish and Redshaw [13] highlighted the importance of social support in mediating these experiences. Women who lacked practical and emotional assistance reported greater difficulty caring for themselves nutritionally, which in turn exacerbated feelings of isolation and emotional distress. Related experiences were reported by Xiao et al. [31], who explored mothers’ experiences of breastfeeding in the early postpartum period. Women described feeding practices as emotionally demanding and closely connected to expectations of maternal competence. Difficulties maintaining regular eating routines while managing infant feeding and household responsibilities were frequently associated with fatigue and emotional strain.

## 4. Discussion

Across the included studies, women described food and eating practices as closely connected to care, responsibility, emotional wellbeing, and expectations surrounding motherhood. The findings reinforce and extend earlier sociological and feminist scholarship on food and care by illustrating how contemporary mothers continue to navigate deeply gendered expectations surrounding self-sacrifice and responsibility. The consistency of themes across multiple studies and contexts, together with the depth of qualitative data, supports the credibility of these findings, although some variation in methodological quality should be taken into account when interpreting the results.

Classic qualitative work by DeVault [9] conceptualised feeding as a form of caring labour that is often invisible and taken for granted. The studies included in this review confirm that such dynamics remain highly relevant during the perinatal period. Women consistently prioritised the nutritional needs of their infants and families over their own, frequently framing this self-denial as an integral component of good motherhood. This observation is consistent with emerging conceptual perspectives suggesting that eating practices may be shaped not only by pleasure or personal preference but also by perceived obligations, caregiving responsibilities and functional self-maintenance [33]. This aligns with Hays’ theory of intensive mothering, in which mothers are expected to devote extensive emotional and physical resources to their children, often at personal cost.

The findings of the present review further support and extend the theory of intensive mothering by demonstrating that nutrition represents a key domain through which maternal responsibility is enacted, monitored and evaluated. Across diverse contexts, women described experiencing pressure to make “correct” dietary choices not only for their own wellbeing but also for the perceived health and future outcomes of their children. These findings suggest that food-related practices function as an important mechanism through which contemporary expectations of “good motherhood” are constructed and reinforced.

The moralisation of food practices observed in recent studies echoes Lupton’s [7] earlier arguments regarding food as a site of moral judgement and self-surveillance. Evidence from qualitative studies such as Sui et al. [32] further illustrates how dietary practices during pregnancy become closely intertwined with concerns about body regulation and gestational weight gain. Women often described monitoring their eating behaviours not only for health reasons but also in response to perceived expectations about appropriate maternal conduct. Across several studies, women described feeling responsible for monitoring their eating behaviours in ways that were closely tied to expectations of being a “good” mother. However, contemporary qualitative evidence suggests that these pressures may be intensified by the proliferation of nutritional guidelines, digital health information, and social media discourse. Women are now exposed to a constant flow of often contradictory messages about what constitutes appropriate maternal nutrition, increasing anxiety and undermining confidence in their own embodied knowledge. Recent qualitative studies have similarly shown that women frequently navigate multiple formal and informal sources of nutritional information during pregnancy, often describing uncertainty regarding which recommendations are trustworthy, culturally appropriate or realistically achievable within the context of everyday maternal life [34].

Women’s experiences of food and dietary decision-making were also shaped by practical constraints, family relationships, financial pressures, and interactions with healthcare professionals. This suggests that nutritional practices during pregnancy and early motherhood cannot be understood solely as individual lifestyle choices. Economic constraints, cultural norms, and healthcare interactions collectively shape women’s capacity to engage in self-care through nutrition. Recent qualitative studies have similarly shown that women’s nutritional practices during pregnancy are strongly influenced by family dynamics, practical support, financial limitations and access to consistent dietary guidance, highlighting the relational and structural dimensions of maternal nutrition [35,36]. This finding resonates with feminist critiques of individualised health responsibility, which argue that public health discourses often obscure the social conditions under which health behaviours occur.

The relationship between nutrition and maternal mental health, while underexplored, emerged as a critical issue. Consistent with earlier qualitative studies on motherhood and emotional labour, the findings suggest that difficulties maintaining adequate nutrition may contribute to emotional exhaustion, stress, and feelings of inadequacy. Similarly, qualitative research on early breastfeeding experiences has highlighted the emotional labour associated with maternal feeding practices. In the study by Xiao et al. [31], mothers described balancing their own nutritional needs with the demands of infant feeding, often within contexts characterised by fatigue and limited support. Interestingly, several studies suggested that women often prioritised the nutritional needs of the infant or family over their own dietary needs during the postpartum period. These accounts echo broader findings from this synthesis, suggesting that maternal nutrition during early motherhood is frequently negotiated within competing care responsibilities. Rather than serving as a resource for wellbeing, food practices can become another domain in which women feel they are failing to meet idealised standards of motherhood. Recent research has also suggested that maternal experiences of food insecurity and nutritional strain may be closely associated with emotional distress, anxiety and perceived caregiving burden, particularly among women already facing social or economic vulnerability [37]. Emerging evidence from nutritional and psychosocial health research further supports the close interrelationship between nutrition and emotional wellbeing, suggesting that dietary patterns may influence psychological functioning through complex biological, behavioural and psychosocial pathways [38].

The findings of this review suggest that maternal nutrition should be approached within the broader social and emotional context of pregnancy and early motherhood. However, the qualitative evidence base remains relatively limited, and the studies included in this synthesis varied in their socio-cultural contexts and sample characteristics.

Future research could further explore how structural factors such as socio-economic inequalities, migration status and access to food environments shape maternal nutritional practices. While the findings of this review primarily highlight the social, emotional and relational dimensions of maternal nutrition, they also have important implications for clinical practice. The experiences described by women suggest that nutritional care during pregnancy and early motherhood should not be limited to the provision of dietary recommendations alone but should also acknowledge the broader social contexts within which nutritional decisions are made. The following examples illustrate how evidence-based nutritional guidance may be integrated with woman-centred care approaches that recognise women’s everyday realities and caregiving responsibilities. Some women described nutritional recommendations as difficult to apply within the realities of daily caregiving and limited support. At the same time, nutritional guidance during pregnancy also requires appropriate clinical individualisation based on maternal health status and obstetric history. Certain maternal conditions or previous pregnancy outcomes necessitate targeted dietary counselling and specific micronutrient supplementation. For example, women with a previous pregnancy affected by a neural tube defect are advised to consume a higher dose of folic acid (4 mg/day) during the periconceptional period and early pregnancy in order to substantially reduce the risk of recurrence. Clinical guidelines indicate that such high-dose folic acid supplementation should begin at least one month before conception and continue through the first trimester [39]. Further examples illustrate the importance of individualising nutritional guidance in pregnancy. Women diagnosed with gestational diabetes mellitus are typically advised to follow structured dietary strategies focusing on carbohydrate distribution and glycaemic control in order to support optimal maternal glucose levels and reduce adverse perinatal outcomes. Similarly, iron-deficiency anaemia during pregnancy requires targeted dietary counselling combined with iron supplementation to restore maternal iron stores and support fetal development. Women with a history of bariatric surgery represent another group requiring specialised nutritional monitoring, as they may be at increased risk of micronutrient deficiencies, including vitamin B12, folate and iron. Together, these examples suggest that nutritional guidance is more effective when clinical recommendations are considered alongside women’s everyday experiences and care responsibilities [40,41,42]. Integrating these evidence-based recommendations within supportive, woman-centred nutritional counselling may help bridge biomedical risk-reduction strategies with women’s lived experiences of food and care during the transition to motherhood. Instead, a more holistic, woman-centred approach is needed, one that recognises nutrition as a social, emotional, and relational practice embedded within the broader transition to motherhood.

These findings suggest that nutritional guidance during pregnancy and early motherhood may benefit from approaches that recognise the social and emotional dimensions of food practices, rather than focusing solely on behavioural compliance.

### 4.1. Implications for Nursing Practice and Policy

The findings of this review highlight the importance of adopting more holistic and woman-centred approaches to nutritional support during pregnancy and early motherhood. Nutritional guidance should not focus exclusively on behavioural compliance or biomedical risk reduction, but also acknowledge the emotional, relational and socio-cultural dimensions of women’s food practices [7,13,34]. Midwives, nurses and other maternity care professionals are well positioned to provide supportive and non-judgemental nutritional counselling that takes into account women’s caregiving responsibilities, emotional wellbeing and everyday realities [16,34]. In addition, continuity of care and individualised nutritional guidance may help women navigate conflicting dietary information and reduce feelings of anxiety, guilt and self-surveillance associated with maternal food practices.

Where appropriate, maternity care professionals may also consider screening for food insecurity and facilitating referral to relevant support services, including dietitians and mental health professionals, in order to provide more comprehensive nutritional and psychosocial support [16,34,35,37].

### 4.2. Strengths and Limitations

This review has several strengths. It synthesises qualitative evidence from diverse socio-cultural contexts and provides an integrated understanding of how women experience nutritional practices during pregnancy and early motherhood. The review also contributes to the existing literature by examining maternal nutrition beyond biomedical perspectives and by highlighting the emotional, relational and identity-related dimensions of food practices during the transition to motherhood.

However, some limitations should also be acknowledged. The included studies varied in methodological approaches, participant characteristics and cultural settings, which may influence the transferability of findings. Only studies published in English were included, potentially limiting the inclusion of relevant evidence from other linguistic contexts. In addition, although the review aimed to capture contemporary qualitative evidence, the number of eligible studies remained relatively limited. Finally, as with all qualitative syntheses, the interpretation of themes may have been influenced by the interpretative nature of thematic synthesis.

### 4.3. Recommendations for Future Research

Future qualitative research could further explore how maternal nutritional practices are shaped by broader structural and social determinants, including socio-economic inequalities, migration experiences, food insecurity and access to maternity care services [28,35,36,37]. Additional research is also needed to examine women’s experiences across more diverse cultural settings and family structures. Furthermore, longitudinal qualitative studies may provide deeper insight into how women’s relationships with food, self-care and maternal identity evolve throughout pregnancy and the extended postpartum period. Future studies could also explore how healthcare systems and maternity services may better support women’s nutritional wellbeing in ways that are both clinically appropriate and emotionally supportive.

Future longitudinal qualitative studies following women from pregnancy through the first two years postpartum could provide a more detailed understanding of how nutritional practices, self-care behaviours and maternal identity evolve over time. Particular attention should be given to populations that remain underrepresented in the current evidence base, including migrant women, women experiencing food insecurity, adolescent mothers, women from ethnic minority communities and those living in socio-economically disadvantaged circumstances. Further research could also explore how nutritional experiences differ across diverse family structures and caregiving arrangements. Such evidence may help inform more equitable and culturally responsive nutritional support strategies during the transition to motherhood.

## 5. Conclusions

The studies included in this review suggest that women’s nutritional practices during pregnancy and early motherhood are closely connected to caregiving responsibilities, emotional wellbeing, social expectations, and access to support. Across different settings, women often described difficulties balancing nutritional recommendations with the practical demands of everyday maternal care. These findings may reflect the broader social expectations and responsibilities placed on women during the transition to motherhood. While nutrition is commonly framed as a health behaviour, women experience it as a moral and emotional practice closely tied to their sense of self as mothers. Recognising these dimensions is essential for developing maternal health strategies that support not only physical outcomes but also women’s autonomy, wellbeing, and mental health during this critical life transition. However, these conclusions should be interpreted with consideration of the limited number of included studies, their diverse socio-cultural contexts and the restriction to English-language publications, which may affect the transferability of the findings. These findings also highlight the importance of relational and context-sensitive nutritional support within maternity care, including approaches that acknowledge women’s lived realities, caregiving demands and emotional wellbeing throughout pregnancy and early motherhood.

## Figures and Tables

**Table 1:** Key characteristics of included qualitative studies on maternal nutrition during the transition to motherhood.

Author (Year)	Country	Study Design	Participants	Data Collection	Contributing Theme(s)	Key Focus
Blau et al. (2020) [27]	USA	Qualitative	Pregnant women	Interviews	Nutrition as self-care and emotional regulation	Food cravings and dietary experiences in pregnancy
Olander et al. (2012) [8]	UK	Qualitative	Pregnant women	Interviews	Loss of autonomy, surveillance, and moral regulation	Dietary advice and perceived autonomy
Marshall et al. (2026) [28]	UK	Qualitative	Pregnant and postpartum women	Interviews	Loss of autonomy, surveillance, and moral regulation; Nutritional practices and maternal mental well-being	Food insecurity during pregnancy
Murray-Davis et al. (2019) [29]	Canada	Qualitative (grounded theory)	Postpartum women	Interviews	Nutrition, identity, and the performance of “good motherhood”	Nutrition and postnatal body image
Bathula et al. (2024) [30]	India	Qualitative	Postpartum women	Interviews/FGDs	Nutrition as self-care and emotional regulation; Nutrition, identity, and the performance of “good motherhood”	Cultural postpartum food practices
Faria-Schützer et al. (2018) [22]	Brazil	Qualitative	Postpartum women with obesity	In-depth semi-directed interviews	Nutritional practices and maternal mental well-being	Eating behaviours, emotional eating and psychological experiences during the postpartum period
McLeish & Redshaw (2017) [13]	UK	Qualitative	Mothers	Interviews	Nutritional practices and maternal mental well-being	Support and emotional wellbeing
Delgado-Pérez et al. (2021) [23]	Spain	Qualitative	Postpartum women, partners and healthcare specialists	In-depth interviews/discussion groups/online forum	Nutrition, identity, and the performance of “good motherhood”	New motherhood, maternal identity, social judgement and healthcare support
Xiao et al. (2020) [31]	China	Qualitative exploratory	Postpartum mothers	Interviews	Nutritional practices and maternal mental well-being; Nutrition, identity, and the performance of “good motherhood”	Breastfeeding and maternal feeding experiences
Sui et al. (2013) [32]	Australia	Qualitative	Pregnant women	Interviews	Loss of autonomy, surveillance, and moral regulation; Nutrition, identity, and the performance of “good motherhood”	Body image, diet and gestational weight gain

**Table 2:** Characteristics of the populations and settings of the included qualitative studies.

Study	Sample Size	Maternal Stage	Participant Characteristics	Cultural/Contextual Setting
Blau et al. (2020) [27]	Eight groups with 68 pregnant women (second trimester)	Pregnancy	Pregnant women receiving prenatal care	USA; prenatal care setting, University of North Carolina–Chapel Hill
Olander et al. (2012) [8]	Four groups; two with prenatal women (n = 9) and two with postnatal women (n = 14)	Pregnancy and postnatal period	Prenatal women (n = 9) and postnatal women (n = 14)	UK; focus groups exploring acceptable healthy eating support services
Marshall et al. (2026) [28]	(n = 11) (second and third trimester)	Pregnancy and postpartum period	Women experiencing or discussing food insecurity around pregnancy	UK; food insecurity examined through a socio-ecological perspective
Murray-Davis et al. (2019) [29]	Eight focus groups (n = 28 women; 4–6 months postpartum)	Early postpartum [4–6 months post-delivery]	Postpartum women, 4–6 months after birth	Canada; nested qualitative study within a pregnancy nutrition/exercise trial
Bathula et al. (2024) [30]	15 in-depth interviews and 2 focus group discussions	Postpartum period [first 6–10 weeks of delivery]	Postnatal mothers and caregivers	India; Government Medical College, community-based postpartum setting; culturally shaped postpartum dietary practices and restrictions
Faria-Schützer et al. (2018) [22]	n = 16 women	Postpartum period	Postpartum women with obesity	Brazil; qualitative study conducted at the University of Campinas (UNICAMP), exploring eating behaviours and emotional experiences during the postpartum period
McLeish & Redshaw (2017) [13]	n = 47 women	Pregnancy and early parenthood	Mothers who had received organised peer support	UK; mainly disadvantaged Black and ethnic minority women, including recent migrants
Delgado-Pérez et al. (2021) [23]	n = 36 women	Early postpartum (first 6 months)	Postpartum women	Spain; qualitative study exploring women’s experiences of new motherhood, self-demand and social expectations during the transition to motherhood
Xiao et al. (2020) [31]	n = 22 women	Early postpartum period	Mothers within the first 6 weeks after delivery	China; breastfeeding and maternal feeding experiences in Shenzhen
Sui et al. (2013) [32]	n = 442 women	Pregnancy	Pregnant women with overweight or obesity	Australia; body image, diet and gestational weight gain

## Data Availability

No new data were created or analyzed in this study. Data sharing is not applicable to this article. The search strategies, PRISMA checklist and qualitative appraisal tables supporting this review are available in the Supplementary Materials.

## Supplementary Materials

The following supporting information can be downloaded at: https://www.mdpi.com/article/10.3390/nursrep16070234/s1, Supplementary File S1: Detailed search strategies for all databases; Supplementary File S2: PRISMA 2020 checklist; Supplementary File S3: CASP qualitative appraisal of included studies.

## Public Involvement Statement

No public involvement in any aspect of this research.

## Guidelines and Standards Statement

Τhis manuscript was drafted in accordance with the Preferred Reporting Items for Systematic Reviews and Meta-Analyses (PRISMA) 2020 Statement for reporting systematic reviews [43].
