# Supplementary material for: Nutritional Practices During the Transition to Motherhood: A Systematic Qualitative Review
Source: Nurs Rep. 2026 Jul 6;16(7):234. doi: 10.3390/nursrep16070234 (PMC13415110; doi:10.3390/nursrep16070234)
Supplement: Supplementary file 1 [file nursrep-16-00234-s001.zip › Supplementary_File_S1_Search_Strategies.pdf]

Supplementary File S1

Detailed Search Strategies for all databases

The electronic search strategy was developed to identify qualitative studies exploring women's experiences of nutrition, food practices and eating behaviours during pregnancy, the postpartum period and early motherhood. Searches were conducted in PubMed, Scopus, Web of Science and CINAHL. The final search update was performed in June 2026.

PubMed

("Pregnancy"[MeSH] OR "Postpartum Period"[MeSH] OR pregnancy OR postpartum OR "transition to motherhood" OR "early motherhood" OR "new mothers") AND ("Nutrition"[MeSH] OR "Diet"[MeSH] OR nutrition OR diet OR "food practices" OR eating OR feeding) AND ("Qualitative Research"[MeSH] OR qualitative OR interviews OR "focus groups" OR ethnograph*)

Scopus

(pregnancy OR postpartum OR "transition to motherhood" OR "early motherhood" OR "new mothers") AND (nutrition OR diet OR "food practices" OR eating OR feeding) AND (qualitative OR interviews OR "focus groups" OR ethnograph*)

Web of Science

TS=(pregnancy OR postpartum OR "transition to motherhood" OR "early motherhood" OR "new mothers") AND TS=(nutrition OR diet OR "food practices" OR eating OR feeding) AND TS=(qualitative OR interviews OR "focus groups" OR ethnograph*)

CINAHL

(MH "Pregnancy+" OR MH "Postpartum Period+" OR pregnancy OR postpartum OR "transition to motherhood" OR "early motherhood") AND (MH "Nutrition+" OR MH "Diet+" OR nutrition OR diet OR "food practices" OR eating) AND (MH "Qualitative Studies+" OR qualitative OR interviews OR "focus groups" OR ethnograph*)

Limits applied: English language; peer-reviewed studies; publication period January 2010 to June 2026.
