# Supplementary material for: Nutritional Practices During the Transition to Motherhood: A Systematic Qualitative Review
Source: Nurs Rep. 2026 Jul 6;16(7):234. doi: 10.3390/nursrep16070234 (PMC13415110; doi:10.3390/nursrep16070234)
Supplement: Supplementary file 1 [file nursrep-16-00234-s001.zip › Supplementary_File_S3_CASP_Table.pdf]

Supplementary File S3

CASP Qualitative Appraisal of Included Studies

Methodological quality was assessed using the Critical Appraisal Skills Programme (CASP) checklist for qualitative research. Studies were appraised in relation to clarity of aims, appropriateness of qualitative methodology, recruitment strategy, data collection, reflexivity, ethical considerations, rigor of analysis and value of findings.

Study	Clear aims	Appropriate methodology	Data analysis rigor	Overall appraisal
Blau et al. (2020)	Yes	Yes	Moderate–High	High quality
Olander et al. (2012)	Yes	Yes	Moderate	Moderate–High quality
Marshall et al. (2026)	Yes	Yes	High	High quality
Murray-Davis et al. (2019)	Yes	Yes	High	High quality
Bathula et al. (2024)	Yes	Yes	Moderate	Moderate–High quality
Chang et al. (2016)	Yes	Yes	Moderate	Moderate quality
McLeish & Redshaw (2017)	Yes	Yes	High	High quality
Faircloth (2014)	Yes	Yes	Moderate	Moderate–High quality
Xiao et al. (2020)	Yes	Yes	High	High quality
Sui et al. (2013)	Yes	Yes	Moderate	Moderate quality

No studies were excluded on the basis of methodological quality. Quality appraisal informed the interpretation of findings and the assessment of confidence in the thematic synthesis.
